# Impact of alcohol consumption, substance use, and smoking on treatment outcomes in tuberculosis: a systematic review and meta-analysis

**DOI:** 10.1186/s13643-025-02888-y

**Published:** 2025-07-05

**Authors:** Bahram Heshmati, Sanaz Omidi, Younes Mohammadi

**Affiliations:** 1 Department of Epidemiology, School of Public Health, Hamadan University of Medical Sciences, Hamadan, Iran; 2 Student Research Committee, Hamadan University of Medical Sciences, Hamadan, Iran; 3 Social Determinants of Health Research Center, Hamadan University of Medical Sciences, Hamadan, Iran

**Keywords:** Treatment outcome, Alcohol consumption, Smoking, Substance use, Tuberculosis

## Abstract

**Background:**

This study aimed to elucidate the influence of alcohol, smoking, and substance use on tuberculosis (TB) treatment failure using a meta-analysis approach.

**Method:**

A comprehensive search strategy was developed and applied to three major databases: MEDLINE, Web of Science, and Scopus. Additionally, Google Scholar, and Google were used to locate grey literature. Studies were identified through title and abstract screening, followed by a full-text review for eligibility. The Newcastle–Ottawa Scale checklist was employed to assess the quality of included studies. Pooled odds ratios (OR) with 95% confidence intervals (CI) were calculated for each factor.

**Results:**

The initial database search and other sources yielded 10,518 articles. After applying inclusion criteria, 19 studies with a total of 180,119 participants were selected for the meta-analysis. The results revealed significant associations between all three factors and treatment failure. Pooled ORs indicated that alcohol consumption (OR 2.05; 95% CI 1.65 to 2.55), smoking (OR = 1.85; 95% CI 1.44 to 2.37), and substance use (OR 2.04; 95% CI 1.63 to 2.55) were each associated with an increased risk of TB treatment failure. Additionally, the majority of included studies demonstrated high methodological quality.

**Conclusion:**

Our findings suggest that alcohol, smoking, and substance use are significant risk factors for unsuccessful TB treatment. To enhance TB treatment efficacy, preventive interventions aimed at reducing these behaviors before treatment initiation are recommended.

**Supplementary Information:**

The online version contains supplementary material available at 10.1186/s13643-025-02888-y.

## Background

Tuberculosis (TB) is a chronic infectious disease caused by the Mycobacterium tuberculosis. It primarily affects the lungs, but can also disseminate to other organs and tissues, including the intestines, meninges, bones and joints, lymph nodes, and skin [1]. TB remains a significant public health threat worldwide. An estimated one-third of the global population is latently infected with TB, meaning they carry the bacteria but do not exhibit symptoms. Among these latent infections, approximately 5 to 10% will progress to active TB disease at some point in their lifetimes [2]. An estimated global total of 10.8 million people fell ill with TB in 2023, equivalent to 134 incident cases per 100,000 populations. The burden of the disease is disproportionately felt in low- and middle-income countries. Regions like Southeast Asia and Africa experience the highest incidence rates. India and China are among the countries with the most significant TB burdens. Globally in 2023, TB caused an estimated 1.25 million deaths [2].

Despite the substantial global burden imposed by TB, the disease is fortunately both curable and preventable. Isoniazid remains the cornerstone of TB preventive treatment for individuals with latent TB infection who are at high risk of progression to active TB disease. The recommended duration of isoniazid prophylaxis is typically at least 6 months [3]. While screening and treatment of individuals with active TB are crucial components, a successful TB control strategy necessitates a multi-pronged approach [4]. However, treatment failure of TB remains a significant threat, as it can lead to drug-resistant strains that are more difficult and expensive to treat [5], treatment failure of TB is defined as the presence of a positive sputum smear in a patient after the initiation of anti-TB treatment [6]. The morbidity and mortality rates are higher in patients with treatment failure compared to those without successful treatment [7].

Studies have identified several factors contributing to TB treatment failure, including age, sex, poverty, pregnancy, comorbidities, and low educational attainment [8, 9]. The influence of alcohol, smoking, and substance use on TB treatment failure is a recognized area of concern. While a definitive causal link remains to be established, accumulating evidence suggests these factors may significantly impact treatment outcomes.

Research findings regarding this association are not entirely homogeneous. Some studies have demonstrated a statistically significant correlation between alcohol consumption, smoking, and substance use with increased risk of treatment failure. However, inconsistencies exist across studies, highlighting the need for further investigation to elucidate the underlying mechanisms and establish definitive cause-and-effect relationships [10–12]; others have not shown such an association [13–15]. One promising approach to address the inconsistencies observed in research on the association between alcohol, smoking, substance use, and TB treatment failure is meta-analysis. This statistically rigorous methodology allows researchers to systematically combine data from multiple studies investigating the same question. By pooling effect sizes from various studies, a meta-analysis can provide a more robust and precise estimate of the overall relationship between these factors and treatment failure [16]. Given the inconsistencies in existing research, this study employed a meta-analysis approach to generate a more robust and generalizable estimate of the association between alcohol, smoking, substance use, and tuberculosis treatment failure.

## Materials and methods

This study was prepared and written based on the PRISMA (Preferred Reporting Items for Systematic Reviews and Meta-Analyses) checklist 2020 (Supplementary material 1) [17].

### Protocol and registration

The protocol of this systematic review and meta‐analysis study has not been registered in any database.

### Eligibility criteria

We employed the PICOS framework to define the eligibility criteria for included studies regardless of age, sex, race, ethnicity, and geographical region [2]. P (population): Individuals diagnosed with TB undergoing anti-tuberculosis treatment. I (exposure): alcohol consumption, smoking, or substance use. C (comparison): individuals with TB undergoing anti-tuberculosis treatment who did not report alcohol consumption, smoking, or substance use. O (outcomes): treatment failure as defined by the included study. S (study design): observational studies including cross-sectional, case–control, and cohort designs. Qualitative studies, trials, case reports, and case series were all excluded. Further, studies from which it was not possible to extract related data and studies with specific populations were excluded.

### Information sources and search

A comprehensive search strategy was implemented to identify relevant studies. We searched three major electronic databases, including Web of Science, MEDLINE (via PubMed), and Scopus, through July 2024. Additionally, Google Scholar and Google were used to locate grey literature such as thesis, dissertations, government reports, unpublished studies, and conference proceedings. Finally, reference lists of retrieved articles were hand-searched to identify potentially relevant studies not captured in the electronic database searches. The terms related to Mycobacterium tuberculosis, alcohol consumption, smoking, and substance use were combined based on Medical Subject Headings (MeSH) and keywords. The exemplary search strategy for PubMed is provided in Supplementary material 1.

### Study selection

Retrieved citations were imported into EndNote software to manage duplicates. Two independent reviewers (B.H and T.O) screened titles and abstracts based on the eligibility criteria. Disagreements were resolved through discussion with a third reviewer (Y.M). Full texts of potentially relevant studies were retrieved and assessed for final inclusion by the two independent reviewers.

### Data collection

A standardized data extraction form was developed to collect relevant information from included studies. Extracted data included first author name, year of publication, country of study, sample size, study design, population characteristics (age, sex), exposure definitions (alcohol consumption, smoking, substance use), outcome definition (treatment failure), effect size estimates (odds ratio), and corresponding confidence intervals (CIs). The data were extracted by two authors (BH and TO) independently.

### Methodological quality

The Newcastle–Ottawa Scale was used to assess the methodological quality of the included studies. Based on this scale, a maximum of 9 stars was assigned to each study. Studies that received seven or more stars were labeled high-quality, and otherwise, studies were classified as low-quality.

### Heterogeneity and publication bias

The heterogeneity across studies was examined using the chi-square (*χ*2) test and tau-square (τ2) test and was quantified by the *I*^2^ statistic. According to the *I*^2^ value, heterogeneity was classified as low (< 50%), moderate (50–74%), or high (≥ 75%). The possibility of publication bias was explored by the Egger and Begg.

### Summary measures

The effect measure of choice was the odds ratio (OR) with 95% CIs. The results were reported based on a random-effects model. The data were analyzed at a significance level of 0.05 using Stata version 16 (StataCorp., College Station, TX, USA).

### Sensitivity analysis

If the between-study heterogeneity was moderate to high (*I*^2 ^≥ 50%), the source of heterogeneity was investigated using a sequential algorithm.

## Results

### Description of studies

**Fig. 1:**
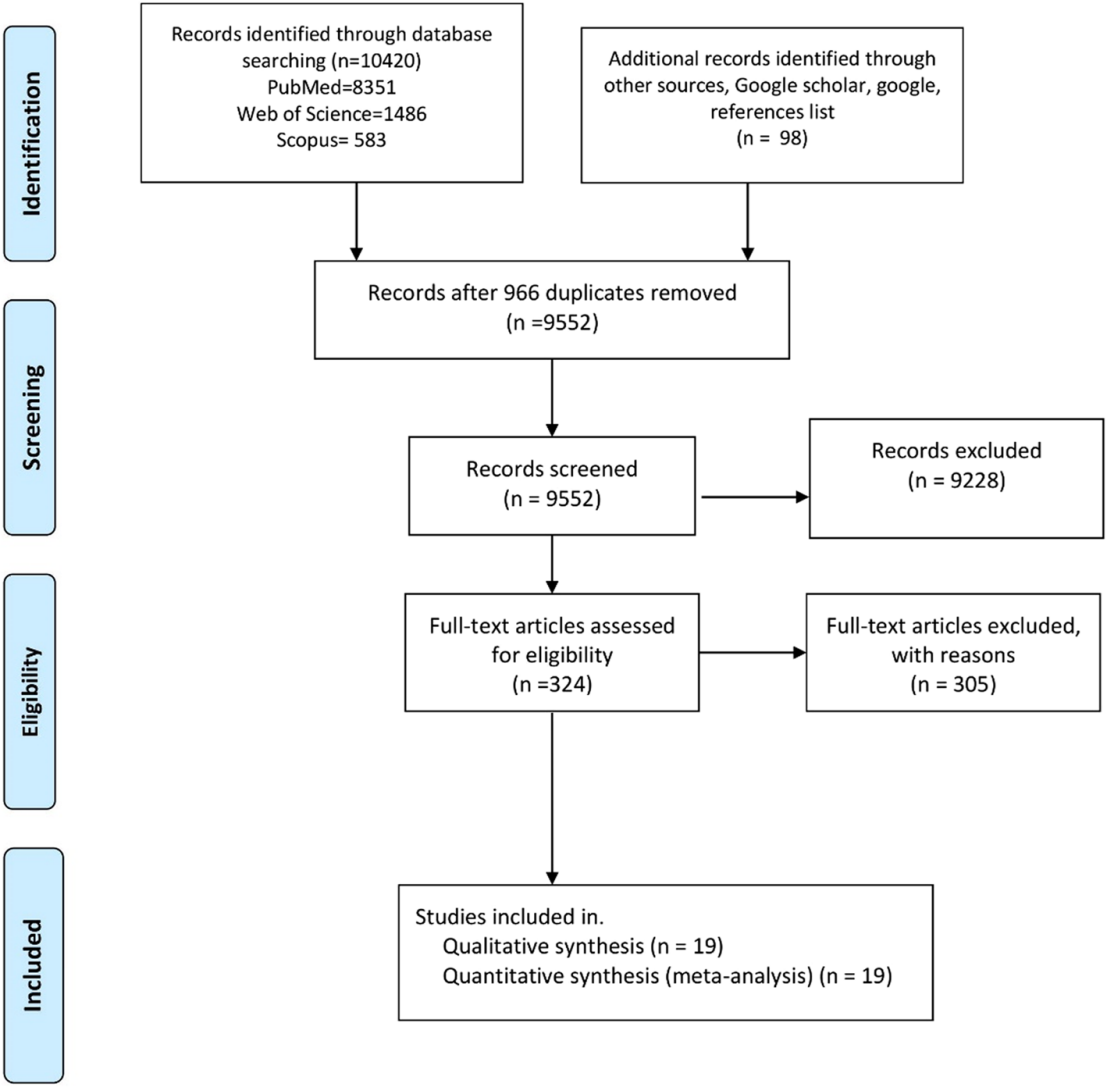
A PRISMA flow chart of the study selection process

Figure 1 shows the PRISMA flow diagram of the study. A total of 10,518 studies were initially retrieved. Duplicate records (*n* = 966) identified across databases were removed. Title and abstract screening excluded 9228 studies deemed irrelevant to the predefined criteria. Following full-text evaluation for eligibility, 19 studies with a total of 180,119 participants were ultimately included in the meta-analysis [13, 15, 18–33].

Table 1 summarizes the characteristics of the included studies. Thirteen studies had high quality and 6 studies had low quality. Cohort studies were the most frequent design (*n* = 10), followed by case–control studies (*n* = 4). Geographically, the studies were distributed across the USA (*n* = 3), China (*n* = 2), India (*n* = 2), and Brazil (*n* = 2). All studies included both men and women, with participant ages ranging from 15 to over 70 years.

**Table 1:** Characteristics of the included studies

Author, year of publication	Country	Age (mean or range	Gender	Study design	Sample size	Quality score (out of eight)	Quality
Aguilar, 2019 [34]	Brazil	45.5	Both	Case–control	284	*8*	High
Balian, 2017 [18]	USA	42	Both	Cohort	992	*6*	Low
Bonacci, 2013 [19]	Mexico	45.4	Both	Cohort	1062	*7*	High
Bongongo, 2020 [30]	South African	> 18	Both	Cross-sectional	180	*8*	High
Albuquerque, 2017 [20]	Brazil	> 19	Both	Cohort	1555	*6*	Low
El-Shabrawy 2016 [21]	Egypt	25–34	Both	Cohort	480	*7*	High
Gegia 2015 [22]	Georgia	> 18	Both	Cohort	524	*7*	High
Lesnic 2016 [15]	Moldova	> 18	Both	Case–control	306	*6*	Low
Veerakumar 2016 [35]	India	> 15	Both	Cross-sectional	235	*7*	High
Miller 2012 [23]	Russia	17–71	Both	Cohort	407	*5*	Low
Montiel 2020 [32]	Paraguay	> 15	Both	Cohort	3034	*8*	High
Oeltmann 2009 [31]	USA	> 15	Both	Cross-sectional	153,268	*7*	High
Sauer 2018 [24]	USA	30–51	Both	Cross-sectional	643	*6*	Low
Sentís 2019 [25]	Portugal	> 15	Both	Cohort	15,478	*7*	High
Serpoosh 2020 [26]	Iran	54	Both	Case–control	286	*8*	High
Singla 2009 [27]	India	14–60	Both	Case–control	118	*7*	High
Tachfouti 2011 [28]	Morocco	35	Both	Cohort	727	*7*	High
Wang 2017 [12]	China	43	Both	Cohort	395	*8*	High
Wobeser 1999 [33]	Canada	45	Both	Cross-sectional	145	*6*	Low

### Effect of alcohol consumption on treatment failure

**Fig. 2:**
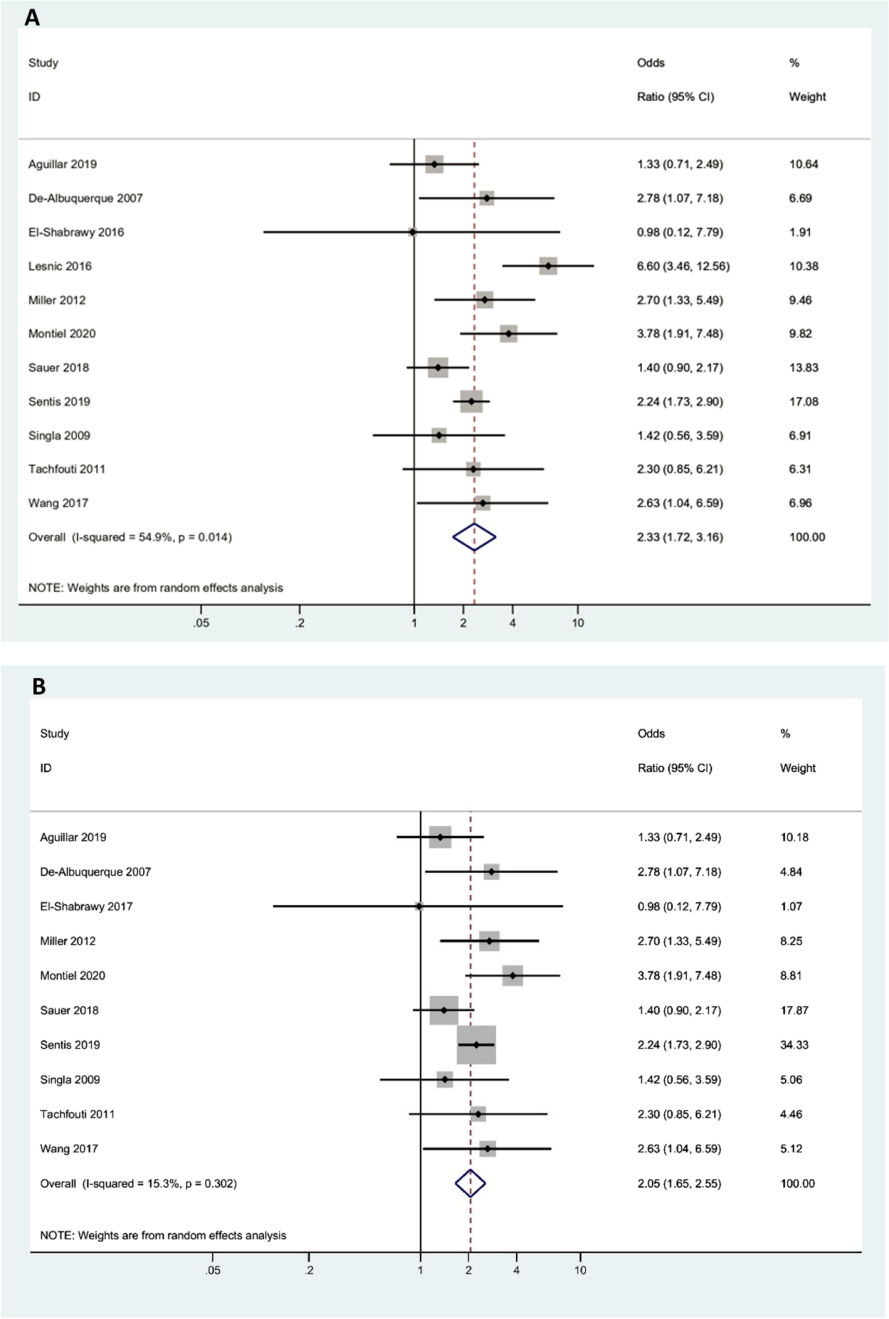
Forest plot illustrating the association between alcohol consumption and treatment failure of tuberculosis. **A** Forest plot of total studies (*I*^2 ^= 54.9%), **B** Forest plot after sensitivity analysis by excluding the Lesnic et al. study [15] (*I*.^2 ^= 15.3%). This shows the significant odds ratio of tuberculosis treatment failure for alcohol users compared to alcohol nonusers. (Publication bias, Egger’s test *P*-value = 0.503)

Eleven studies assessed the association between alcohol consumption and treatment failure. Six studies reported a significant positive association, four reported a positive non-significant association, and one reported a negative non-significant association. The overall odds ratio of treatment failure for patients who consume alcohol versus who do not consume alcohol was 2.33 (95% CI, 1.72 to 3.16). Between-study heterogeneity was moderate (*I*^2 ^= 54.9%) (Fig. 2A). To investigate the source of heterogeneity, sensitivity analysis was done via the on out removed method. By excluding the Lesnic et al. study [15] the result remained significant with a decreased interstudy heterogeneity (OR = 2.05, 95% CI 1.65 to 2.55, *I*^2 ^= 15.3%) (Fig. 2B). There was no evidence of publication bias based on the Begg test (*P* = 0.624) and Egger test (*P* = 0.503).

### Effect of smoking on treatment failure

**Fig. 3:**
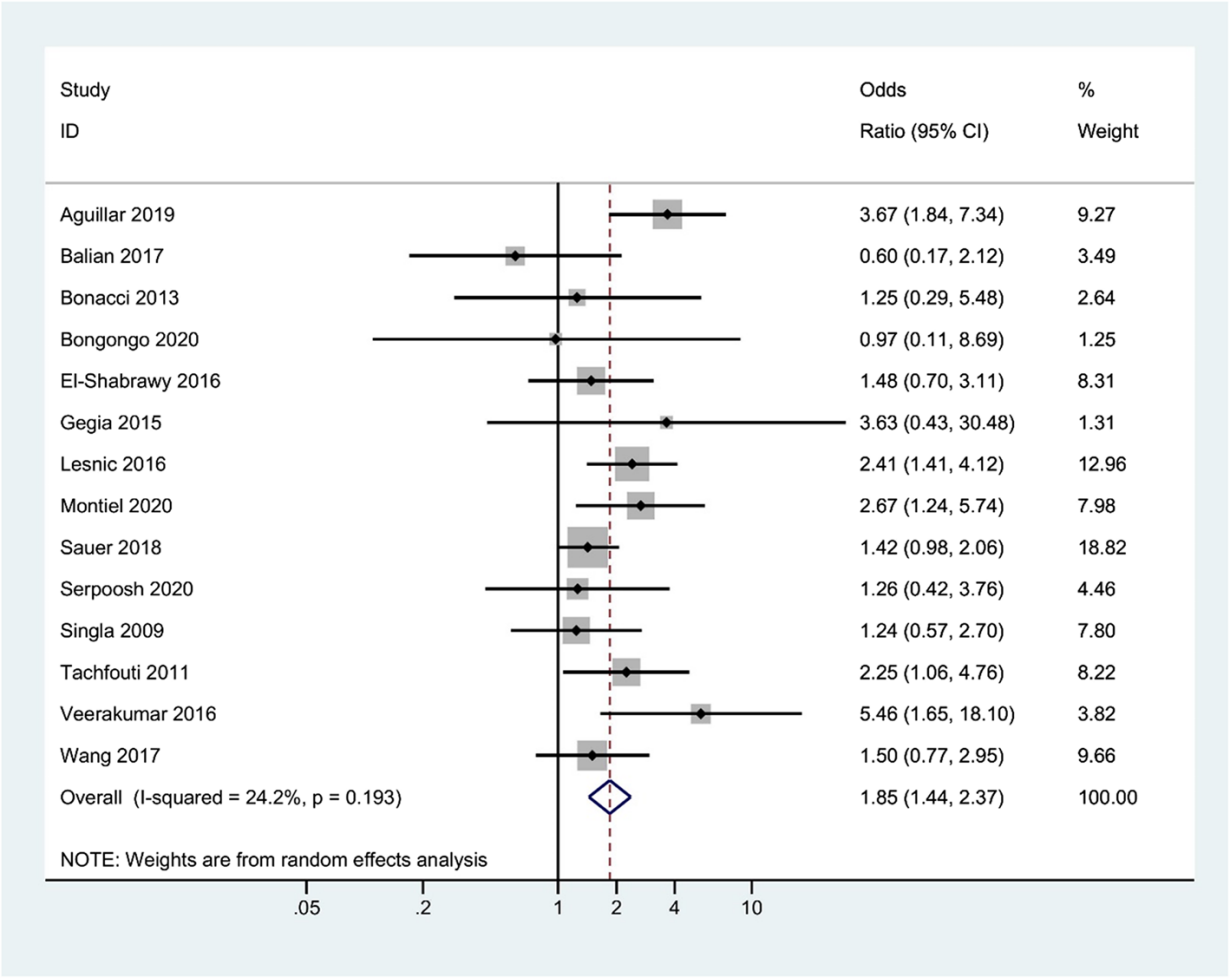
Forest plots illustrating the association between smoking and treatment failure of tuberculosis. This shows the significant odds ratio of tuberculosis treatment failure for smokers compared to non-smokers. (Publication bias, Egger’s test *P*-value = 0.648)

Fourteen studies explored the association between smoking and treatment failure. The overall odds ratio of treatment failure for smoker patients versus non-smoker patients was 1.85 (95% CI, 1.44 to 2.37). Between-study heterogeneity was low (*I*^2 ^= 24.2%). There was no evidence of publication bias based on the Begg test (*P* = 0.624) and Egger test (*P* = 0.503) (Fig. 3). Sensitivity analyses were not conducted, as the level of heterogeneity was low.

### Effect of substance use on treatment failure

**Fig. 4:**
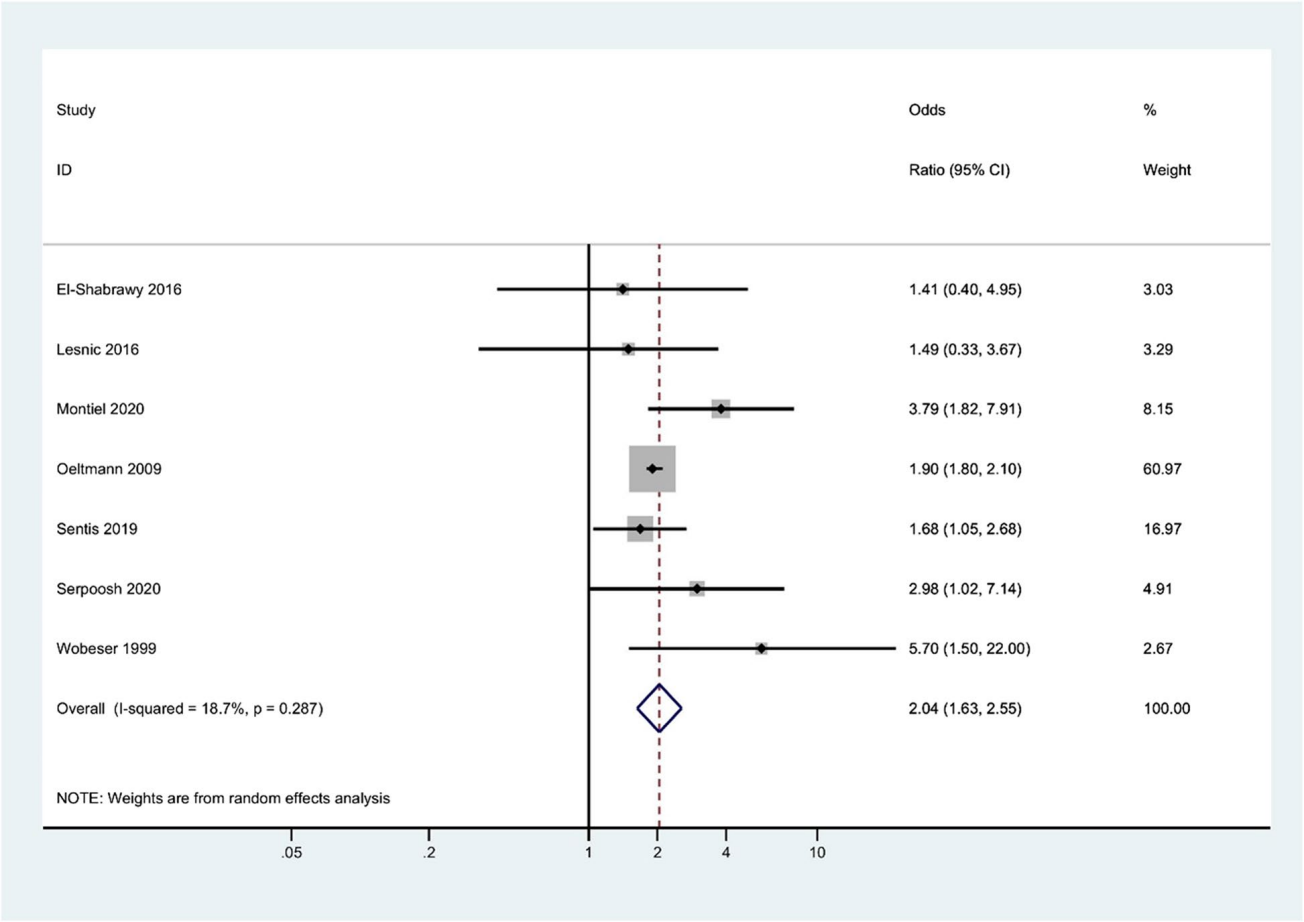
Forest plots illustrating the association between substance use and treatment failure of tuberculosis. This shows the significant odds ratio of tuberculosis treatment failure for substance use compared to nonuser. (Publication bias, Egger’s test *P*-value = 0.212)

Seven studies examined the relationship between substance use and treatment failure. The overall odds ratio of treatment failure for substance user versus non-user was 2.04 (95% CI, 1.63 to 2.55). Between-study heterogeneity was low (*I*^2 ^= 18.7%). There was no evidence of publication bias based on the Begg test (*P* = 1.000) and Egger test (*P* = 0.212) (Fig. 4). Sensitivity analyses were not conducted, as the level of heterogeneity was low.

## Discussion

This meta-analysis aimed to address inconsistencies in the literature regarding the association between alcohol consumption, smoking, and substance use with TB treatment failure. We found statistically significant positive associations between all three factors and treatment failure. Compared to non-user, alcohol user increased the risk of treatment failure by 2.05 times, substance user by 2.04 times, and smoker by 1.85 times. All three factors emerged as significant contributors to treatment failure. These findings highlight the importance of addressing alcohol consumption, substance use, and smoking in TB management programs, particularly in developing countries. The high prevalence of smoking (over 1 billion smokers globally, concentrated in developing regions) and substance use (estimated at 35 million individuals) in these areas underscores the urgency of tackling these modifiable risk factors [36].

Alcohol consumption is a recognized detriment to global public health, directly impacting progress towards achieving several Sustainable Development Goals (SDGs). These include targets related to infectious diseases (HIV, viral hepatitis, tuberculosis), non-communicable diseases, mental health, injuries, and poisonings. Furthermore, the WHO emphasizes that alcohol consumption exacerbates existing health inequalities both between and within countries, hindering progress towards SDG 10, which focuses on reducing these inequalities. A WHO report indicates that in 2019, 56% of the world’s population aged 15 + (65% of females, 48% of males) abstained from drinking alcohol. An estimated 400 million people, or 7% of the world’s population aged 15 years and older, lived with alcohol use disorders [37]. The WHO 2018 global status report on alcohol and health highlights a decline in alcohol consumption across all WHO regions since 2000, with the exceptions of the Western Pacific and South-East Asia Regions, which have a high prevalence of tuberculosis [38].

The significant impact of alcohol consumption, smoking, and substance use on human health, particularly regarding TB, necessitates robust control and prevention strategies for these behaviors in TB patients. Systematic reviews and meta-analyses of observational studies have consistently demonstrated a detrimental association between the global TB epidemic and smoking. Exposure to tobacco smoke has been linked to increased risk of TB infection, active TB disease, and TB mortality. Notably, evidence suggests that mortality from TB is significantly higher among smokers compared to non-smokers. Conversely, studies have shown that smoking cessation can significantly reduce the risk of TB mortality by up to 65% [39].

Studies have identified two key mechanisms by which smoking and substance use contribute to TB burden: immune system dysfunction and treatment non-adherence. Immune system dysfunction: smoking and substance use can impair the immune response through ciliary dysfunction in the respiratory tract. This dysfunction weakens the body’s natural defenses against Mycobacterium tuberculosis infection, increasing susceptibility. Impaired macrophage function due to these substances further reduces immune response. Treatment non-adherence: both smoking and substance use have been negatively associated with adherence to anti-TB treatment regimens. Adherence exceeding 90% is crucial for successful treatment and TB elimination globally. Non-adherence has severe consequences: spread of TB: it can fuel the spread of TB in the population, leading to increased morbidity and mortality. Longer treatment duration: non-adherence often necessitates longer treatment regimens, extending the treatment duration by an estimated 236 days. Delayed diagnosis and advanced disease: substance users may delay seeking treatment, leading to increased infectiousness and advanced disease at diagnosis. Increased costs and treatment challenges: non-adherence can necessitate more complex and expensive combination therapies, which can be further complicated by side effects, potentially leading to poorer treatment outcomes. Progression of latent TB: in individuals with latent TB infection, substance abuse can exacerbate immune dysfunction, increasing the risk of progression to active TB disease [31, 40–42].

Alcohol consumption is a well-established risk factor for TB, with studies indicating a prevalence of approximately 20% among TB patients. A meta-analysis suggests that alcohol consumption is associated with a three-fold increase in the risk of developing TB, potentially contributing to an estimated 17% of all TB incidence cases and 15% of all TB mortality cases globally. Mechanistically, alcohol’s influence on TB treatment failure appears to be two-pronged: immune system impairment: alcohol weakens the immune system, rendering individuals more susceptible to infectious agents like Mycobacterium tuberculosis. This effect manifests through decreased macrophage responsiveness to the bacteria and disrupted cytokine production by monocytes. Increased transmission risk: alcohol consumption patterns, particularly among individuals with alcoholism who frequent bars, shelters, and prisons, can create high-risk environments for TB transmission. Furthermore, studies suggest a potential link between alcohol consumption and an increased risk of developing multidrug-resistant tuberculosis [43–46].

### Implications for medical practice

This study highlights the effect of alcohol consumption, smoking, and substance use on TB treatment failure. These findings emphasize the importance of screening all TB patients for these modifiable risk factors before initiating anti-TB treatment. Early identification allows for interventions to promote smoking cessation and address alcohol and substance use issues. Such interventions can improve treatment outcomes by mitigating the negative effects of these factors on treatment success.

### Strengths of the meta-analysis approach

Compared to individual studies, this meta-analysis offers several advantages: increased precision: by pooling data from multiple studies, this meta-analysis provides a more precise estimate of the true association between smoking, substance use, and TB treatment failure. Reduced bias: it mitigates the limitations of small sample sizes often seen in single studies, reducing the risk of false-negative results. Additionally, by incorporating studies with potentially conflicting findings, meta-analysis can identify true underlying effects and minimize the impact of outliers. Enhanced generalizability: by combining data from studies with diverse populations and sample sizes, this meta-analysis strengthens the external validity (generalizability) of the results, allowing for broader application to clinical practice.

## Limitations and future directions

Despite its strengths, this meta-analysis has limitations: language restriction: limiting the search to English-language studies may have excluded relevant research in other languages. Publication bias: studies with negative or non-significant findings may be less likely to be published, potentially introducing publication bias. Observational design: this meta-analysis focused on observational studies, which have inherent limitations such as confounding variables and difficulties establishing temporality. Ideally, future research could utilize interventional studies to definitively assess the causal effects of smoking and substance use on TB treatment failure.

## Conclusion

This meta-analysis provides strong evidence for a positive association between smoking and substance use with treatment failure in TB patients. Screening for these factors before initiating anti-TB treatment is crucial to improve patient outcomes. Future research should investigate interventions to address smoking, alcohol, and substance use in this population and further explore the causal relationships through interventional studies.

## Supplementary Information

Supplementary Material 1.

## Data Availability

The corresponding author is responsible for data. Access to all relevant raw data will be free to any scientist.

## Abbreviations

TB: Tuberculosis
PRISMA: Preferred Reporting Items for Systematic Reviews and Meta-Analyses
OR: Odds ratio
CIs: Confidence intervals
