# Correction: Impact of alcohol consumption, substance use, and smoking on treatment outcomes in tuberculosis: a systematic review and meta-analysis

**DOI:** 10.1186/s13643-026-03210-0

**Published:** 2026-06-01

**Authors:** Bahram Heshmati, Sanaz Omidi, Younes Mohammadi

**Affiliations:** 1 Department of Epidemiology, School of Public Health, Hamadan University of Medical Sciences, Hamadan, Iran; 2 Student Research Committee, Hamadan University of Medical Sciences, Hamadan, Iran; 3 Social Determinants of Health Research Center, Hamadan University of Medical Sciences, Hamadan, Iran

**Correction: Syst Rev 14, 139 (2025)**

**https://doi.org/10.1186/s13643-025-02888-y**

Following publication of the original article [1]. The authors reported that Figures 2a and 2b were identical on the published article. The original article has been corrected.

**Incorrect Figure 2a**

**Fig. 2:**
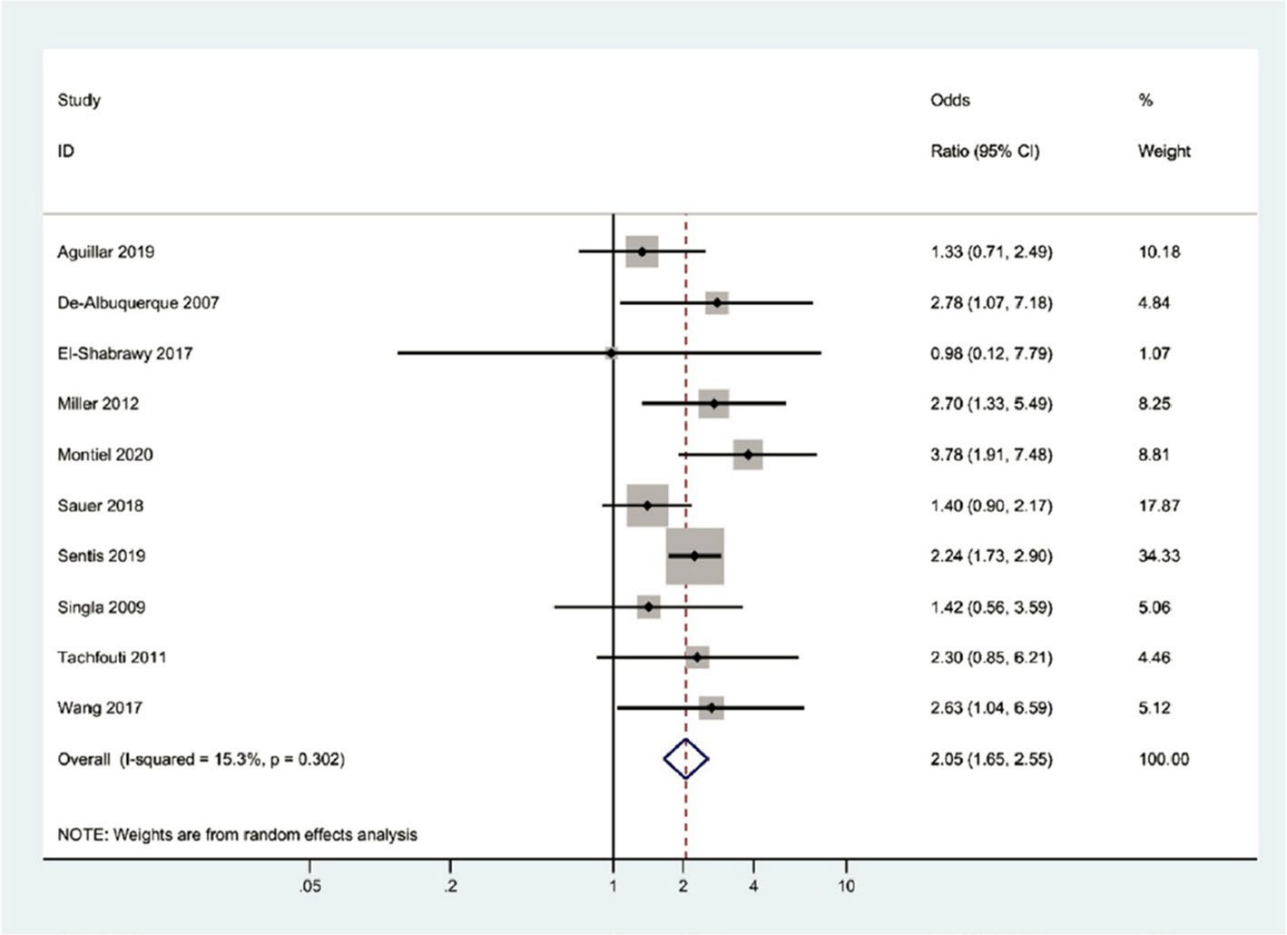
Forest plot illustrating the association between alcohol consumption and treatment failure of tuberculosis. **A** Forest plot of total studies (*I*^2^ = 54.9%), **B** Forest plot after sensitivity analysis by excluding the Lesnic et al. study [15] (*I*.^2^ = 15.3%). This shows the significant odds ratio of tuberculosis treatment failure for alcohol users compared to alcohol nonusers. (Publication bias, Egger’s test *P*-value = 0.503)

**Correct Figure 2a**

**Fig. 2:**
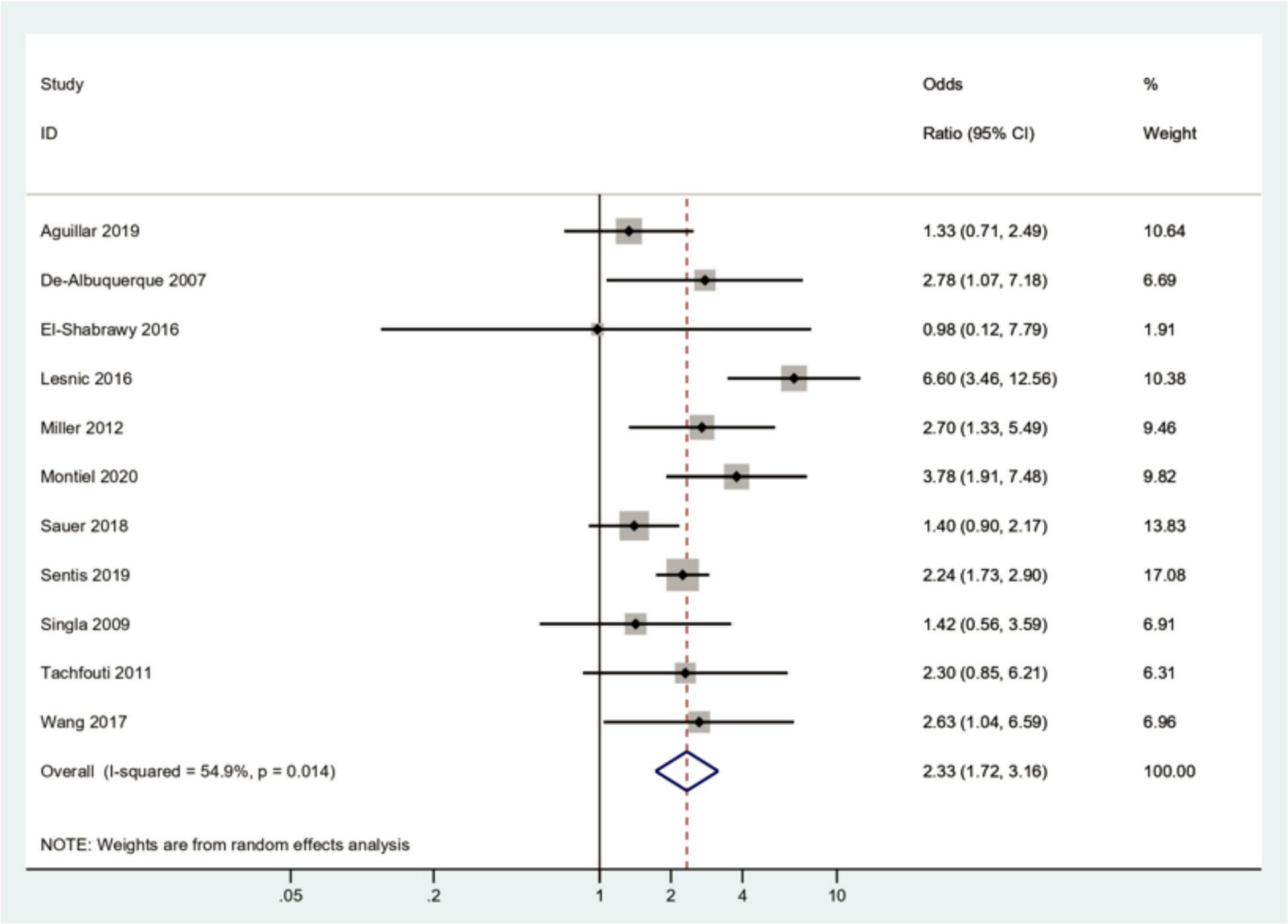
Forest plot illustrating the association between alcohol consumption and treatment failure of tuberculosis. **A** Forest plot of total studies (*I*^2^ = 54.9%), **B** Forest plot after sensitivity analysis by excluding the Lesnic et al. study [15] (*I*.^2^ = 15.3%). This shows the significant odds ratio of tuberculosis treatment failure for alcohol users compared to alcohol nonusers. (Publication bias, Egger’s test *P*-value = 0.503)
